# Validation of the Korean Version of the Nursing Profession Self-Efficacy Scale: A Methodological Study

**DOI:** 10.3390/ijerph18031080

**Published:** 2021-01-26

**Authors:** Jina Oh, Haeryun Cho, Yae Young Kim, So Yeon Yoo

**Affiliations:** 1 College of Nursing, Institute of Health Science, Inje University, Busan 47392, Korea; ohjina@inje.ac.kr; 2 Department of Nursing, Wonkwang University, Iksan 54538, Korea; 3 Department of Nursing, Chung-ang University, Seoul 06974, Korea; yaeyoung@gmail.com; 4 Department of Nursing, Kyungil University, Gyeongbuk 38428, Korea; soyeon.yoo@gmail.com

**Keywords:** nurses, professionalism, questionnaires, self-efficacy, reliability, validity

## Abstract

Background: The Nursing Profession Self-Efficacy (NPSE) scale was developed to reflect the characteristics of nursing tasks. This study was conducted to validate the Korean version of the NPSE (K-NPSE) scale. Methods: The NPSE scale with nineteen items was translated into Korean after forward and backward translation according to Devellis’ guideline. For the exploratory factor analysis (EFA), 298 nurses participated and criterion-related validity and reliability were verified. For the confirmatory factor analysis (CFA), 218 other nurses participated. Content validity, criterion-related validity, and internal consistency reliability were examined. Additionally, construct validity was examined. SPSS and AMOS were used for the data analyses. Results: Nineteen items were selected after evaluating the content and cognitive validity and comprised three factors: “Professional (10 items)”, “Advocating (4)”, and “Caring (5)”. Construct validity was supported by the CFA. Criterion-related validity was supported by comparison with the General Self-Efficacy Test (r = 0.43, *p* < 0.001). Cronbach’s alpha of the K-NPSE was 0.93. Conclusions: Study findings indicate that the K-NPSE could be useful for assessing nurses’ self-efficacy. The K-NPSE may be used as a valuable reference for developing programs or policies that promote nursing professionals. It is expected that continued use of this scale in various clinical settings to further generalize and validate the scale.

## 1. Introduction

Nurses are required to possess multilateral qualities, from in-depth knowledge of nursing, science, technology, and social sciences, to highly-developed critical thinking and decision-making skills [1,2]. In order to support nurses to gain these qualities, it is important to reorganize the nursing system, develop relevant policies aimed at improving working conditions, and implement measures to strengthen the internal motives of nurses [3]. 

Self-efficacy has been considered a key factor that contributes to positive internal changes in nurses [4]. Self-efficacy, introduced by Bandura [5], has been widely used to predict the motives and outcomes of human behaviors. However, general self-efficacy is insufficient for explaining the motives and outcomes of individual performance within specific situations of specific fields [6]. Self-efficacy plays an important role in boosting overall performance and outcomes of professionals, and thus it serves as a key predictor of outcomes in the corresponding profession [7]. Professional self-efficacy also influences work capacity and the behaviors required in unique situations, often determining the quality of decision-making, performances, and accomplishments [6]. Particularly, the quality of care is dependent on a nurse’s competence, and high self-efficacy not only boosts their professional nursing capacity but also contributes to improving the quality of care [3]. 

An individual’s confidence in one’s own ability to coordinate and practice behaviors required to accomplish a task in a professional field may vary widely depending on the profession involved [6,8]. Thus, to evaluate professional self-efficacy, assessment tools specific to the occupation must be designed. Various countries have developed professional self-efficacy scales for teachers [4,8] and counselors [9,10] in an effort to measure the degree of self-efficacy appropriate to each unique profession. Caruso et al. [6]. developed a self-efficacy tool that reflects the characteristics of nursing tasks. The Nursing Profession Self-Efficacy scale (NPSE) was developed in Italy in 2016 and consists of 19 items in two dimensions (characteristics of nursing situations and professional situations). However, several studies on nursing self-efficacy have still used general self-efficacy tools [11,12], a practice which has undermined the accuracy of these studies because they do not measure nursing profession self-efficacy. Therefore, there is a need to validate the specifically designed for the nursing profession for use in each country. 

The nursing environment between Italia and South Korea is similar; arduous working conditions [6], a shortage of nurses, a high nurse-to-patient ration, and outcome-based nursing education [13]. Therefore, the NPSE is a potentially valid instrument that can be used with Korean nurses. 

When developing a tool, it is necessary to verify the validity of the tool in various ways to present reasonable evidence for the use of the tool [14]. It is particularly important to use a more meticulous validation process when using a tool that was developed in a country with sociocultural differences [15]. Thus, this study translated and adapted the NPSE [6] into Korean.

This study aims to establish the validity and reliability of the Korean version of the NPSE (K-NPSE), with the specific objectives: (a) to test the validity and reliability of the K-NPSE, and (b) to modify the scale, creating a K-NPSE that is better suited to Korean nurses.

## 2. Methods

### 2.1. Study Design

This study is a methodological study to validate the K-NPSE originally developed by Caruso et al. [6]. The translation of the tool followed the World Health Organization [16] guidelines and the validation of the tool followed Devellis’ [14] guidelines.

### 2.2. Ethics Consideration 

To ensure participant rights and ethical considerations, this study was approved by the Institutional Review Board at W University (IRB No. WKIRB-201711-SB-082).

### 2.3. Study Procedure 

#### 2.3.1. Translation of Tool 

After obtaining permission from the NPSE developer for the Korean version, the item development of the K-NPSE proceeded in compliance based on the guideline for translation and application of tools [16]. 

At first, the primary translation was independently performed by four Korean-English bilingual nurses, and then expert panels were formed with one professional translator, three nursing professors proficient in English and Korean, and four primary translators, whereupon the panel examined the consistency and accuracy of the four translations by comparing them with the original tool. A reverse translation was followed by a professional translation. Two native English-speaking nursing professors without any prior knowledge of the tool’s development confirmed whether the source sentences and reverse-translated sentences were consistent in meaning. 

#### 2.3.2. Content Validity Test 

Because the content validity should be established by 3–10 experts [17], we formed a six experts panel including two nursing professors, one nursing doctoral candidate, and three head nurses. Primary content validity testing was performed on November, 2017, and the second round of content validity testing was performed 20 days later. Content validity testing by the expert panel was performed using the Item-Content Validity Index (I-CVI) and Scale-Content Validity Index (S-CVI). 

#### 2.3.3. Pilot Survey 

The pilot survey of initial K-NPSE was conducted with 28 nurses. The time it took to complete the survey, comprehension of the item, and appropriateness of the questionnaire structure (font size, questionnaire arrangement, length of item) were rated on a five-point scale. After the survey, an additional interview was conducted to ask about items with unclear meaning due to vague expressions and other difficult-to-understand items (cognitive validity testing) in order to finalize the scale.

#### 2.3.4. Main Survey

##### Study Participants and Setting

Nurses working in 300 beds over hospitals were enrolled nationwide. Nurses with more than one year’s experience were included, but nurses who do not directly provide patient care were excluded. This study targeted 5 hospitals in Seoul and Gyeonggi- province, 3 hospitals in Gyeongsang- province and Gangwon- province, and 2 hospitals in Jeolla-do and Chungcheong-province.

There were separate groups of subjects for the exploratory factor analysis (EFA) and confirmatory factor analysis (CFA). To satisfy the requirement of 200–300 participants for stable testing of the tool’s validity and reliability for the EFA [14,18], the main survey was conducted on 311 nurses, and 298 replies were analyzed. Based on the requirement for a minimum of 200 people for CFA [19], 221 nurses were surveyed in consideration of potential dropped. Finally, 218 questionnaires were analyzed after excluding three for incomplete responses. 

##### Data Collection

Data for EFA and criterion-related validity and reliability testing were collected from January, 2018, and data for CFA were collected from October to November, 2018. Data were collected from 13 hospitals, with 3–5 institutions that were convenience sampled from each of the three regions. After an explanation of the purpose, procedure, and method of the study, the participants signed a written consent form by themselves. The surveys were immediately collected at the site upon completion, and a small gift worth 5 USD was given to all participants. 

#### 2.3.5. Validity Test

Construct validity was tested using EFA and CFA. Principal component analysis and Varimax rotation were used, and model fit, convergent validity, and discriminant validity were analyzed through CFA. Then, the distribution of scores and correlation among survey items were reviewed to analyze the integrity of the items. 

Criterion-related validity was tested by using the general self-efficacy scale, which has the same theoretical background as the K-NPSE [20]. The general self-efficacy scale was used because it is the most common scale for measuring self-efficacy among nurse.

#### 2.3.6. Reliability Test

Internal consistency and split-half reliability of the K-NPSE were analyzed to test reliability. To assess internal consistency, Cronbach’s α value for the entire scale and for each item were computed. In this study, 149 samples were extracted randomly among 298 and tested the split-half reliability using Cronbach’s α to prevent the scale being assessed as highly reliable due to a large sample size [14]. 

### 2.4. Instruments 

#### 2.4.1. Nursing Profession Self-Efficacy

Nursing profession self-efficacy was measured using the NPSE developed by Caruso et al. [6] after approved by developer. The NPSE consists of 19 items in two dimensions including nursing situations (12 items) and professional expertise situations (7). Each item was rated on a five-point Likert scale; a higher score indicates higher nursing profession self-efficacy. The Cronbach’s α for the entire original scale was 0.83 [6]. The Cronbach’s α was 0.92 in this study.

#### 2.4.2. General Self-Efficacy

General self-efficacy was measured using the general self-efficacy scale developed and validated by Kim [20] after approved by developer. This scale consists of 24 items in 3 dimensions including confidence (7 items), self-control efficacy (12), and task difficulty (5). Each item was rated on a six-point Likert scale; a higher score indicates higher self-efficacy. Cronbach’s α for the entire scale and for each of the dimensions were all above 0.80 at the time of development [20]. The Cronbach’s α was 0.83 in this study. 

### 2.5. Data Analysis

The collected data were analyzed using the IBM SPSS Statistics 24 (IBM Inc., Chicago, IL, USA) and IBM SPSS AMOS 24 (IBM Inc., Chicago, IL, USA) software.

1. For the content validity of the scale, each item was computed using I-CVI to select those with a value of 0.80 or higher [17]. S-CVI was tested using S-CVI/Average (Ave) and S-CVI/Universal Agreement (UA) [21].
2. General characteristics of the participants were analyzed using descriptive statistics.
3. The collected data were tested the appropriation of EFA using the Kaiser–Meyer–Olkin (KMO) test and Bartlett’s test of sphericity. For evaluating construct validity, factor analysis was conducted using principal component analysis with Varimax rotation [18,22].
4. The goodness of fit of the model for CFA was analyzed using Chi-square (χ2), standardized Chi-square (χ2/df), Goodness of Fit Index (GFI), Adjusted Goodness of Fit Index (AGFI), Comparative Fit Index (CFI), Normed Fit Index (NFI), Tuker–Lewis Index (TLI), and Root Mean Squared Error of Approximation (RMSEA).
5. Convergent validity was tested using Construct Reliability (CR) and Average Variance Extracted (AVE).
6. Discriminant validity was tested using the square root of AVE and Pearson’s correlation coefficient.
7. Items were analyzed using mean, standard deviation, skewness, kurtosis, Item-Total Correlation (ITC), Cronbach’s α after item removal, and mean and standard deviation by factor.
8. Criterion-related validity was tested using Pearson’s correlation coefficient.
9. Reliability was tested using Cronbach’s α for internal consistency and random split-half reliability of the sample.

## 3. Results

### 3.1. Content Validity Test 

In the first expert content validity test, CVI for 19 preliminary items ranged from 0.71–1.0, with S-CVI/Ave of 0.98 and S-CVI/UA of 0.89. Based on the CVI, none of the items were removed, but some awkward Korean terms and expressions were revised. In the second content validity testing, CVI ranged from 0.86–1.0, with S-CVI/Ave of 0.96 and S-CVI/UA of 0.74. None of the items were removed, but the phrasing of some of the items was polished.

### 3.2. Pilot Survey 

Out of a score of 5, overall item comprehension was 4.11 ± 0.79. The font size (4.36 ± 0.73), survey arrangement (4.29 ± 0.71), and item length (4.11 ± 0.79) were appropriated. In an additional interview to assess cognitive validity, the items were clear and easy to understand.

### 3.3. General Characteristics of Participants 

The demographics of EFA were as follows: about 99.7% of the participants who completed the first survey were women, and 75.2% were single, with a mean age of 28.8 years. The mean clinical career as a nurse was 76.3 months, and the mean career at their current ward was 45.7 months. The greatest number of participants were college graduates (53.4%), and 55.0% had no religion. 

The demographics of CFA were as follows: about 98.2% were women, and 78.0% were single, with a mean age of 29.1 years. The mean clinical career as a nurse was 77.5 months, and the mean career at their current ward was 50.5 months. The majority of the participants (66.3%) had an associate degree, and 48.6% had no religion. 

### 3.4. Construct Validity Test 

**Figure 1:**
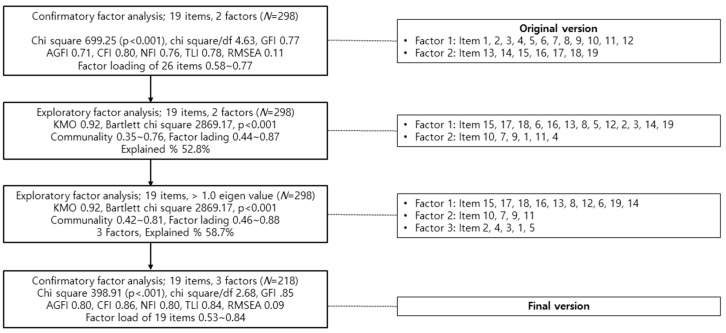
Flow of factor analysis. AGFI = Adjusted Goodness of Fit Index; CFI = Goodness of Fit Index; KMO = Kaiser–Meyer–Olkin; NFI = Normed Fit Index; RMSEA = Root Mean Squared Error of Approximation; TLI = Tuker–Lewis index.

The construct validity test was performed to examine whether the components of the original scale were appropriate for the Korean version (Figure 1). The CFA results were as follows: χ2 699.25 (*p* < 0.001), standardized χ2 4.63, GFI 0.77, AGFI 0.71, CFI 0.80, NFI 0.76, TLI 0.78, and RMSEA 0.11. Two factors were designated for EFA and compared with the structure of the original scale. The items for the two designated factors differed from those in the original scale, so we examined the appropriate composition for the K-NPSE.

Based on the KMO of 0.92 and χ2 = 2869.17 (df = 171, *p* < 0.001) in Bartlett’s test of sphericity, the data were considered suitable for factor analysis [18,22]. Therefore, EFA was performed with an eigenvalue of 1.0 or higher as the cutoff. The communality of the 19 items were all above 0.40, ranging from 0.42 to 0.81. All items had significant descriptive power, and none of the items were deleted. Factor loading ranged from 0.46 to 0.88, thereby satisfying the requirement to be above 0.30 but not close to 1.0. In EFA, three factors were identified with a descriptive power of 58.7%, thereby satisfying the requirement that the descriptive power of a scale should range between 50.0 and 60.0% [23] (Table 1).

Each factor was named “professional situation (factor 1),” “advocating situation (factor 2),” and “caring situation (factor 3).” The goodness of fit for the model was assessed with CFA with these 19 items in three factors, and the fitness indices were the following: χ2 398.91 (*p* < 0.001), standardized χ2 2.68, GFI 0.85, AGFI 0.80, CFI 0.86, NFI 0.80, TLI 0.84, and RMSEA 0.09 (Table 2). All items satisfied the required standardized factor loading (FL) of above 0.50 [18], so all of the items remained. 

The CR for testing the convergent validity of the finalized model ranged from 0.89 to 0.93 with an AVE of 0.51–0.77, thereby satisfying the requirement of a CR of above 0.70 and AVE of above 0.50 [18] (Table 2). The square root of AVE for the discriminant testing was checked for whether it is greater than the correlation coefficients by each factor (Table 3). The square root of AVE ranged from 0.71 to 0.88 and correlation coefficients ranged from 0.62 to 0.66; thus discriminant validity was established. 

### 3.5. Item Analysis

The mean total score for the 19-item K-NPSE was 71.23 ± 8.65. The mean score for each item was 3.43–4.14 with a standard deviation of 0.60–0.81, and there were no outliers. Skewness was −0.40–0.17 and kurtosis was −0.39–0.40, showing that the items were not leaning toward one side. ITC was 0.51–0.68, and Cronbach’s α when each item is deleted was 0.92 (Table 4). 

### 3.6. Criterion-Related Validity

The K-NPSE and general self-efficacy had a significant positive correlation with a correlation coefficient of 0.43, thereby meeting the standard of 0.40–0.80 for establishing criterion-related validity [15,24]. In each dimension, there were significant positive correlations for the professional situation (r = 0.42, *p* < 0.001), advocating situation (r = 0.26, *p* < 0.001), and caring situation (r = 0.41, *p* < 0.001) (Table 3). 

### 3.7. Reliability Testing

The Cronbach’s α for the K-NPSE was 0.93. The Cronbach’s α of each factor ranged 0.82–0.88; professional situation (0.88), advocating situation (0.85), and caring situation (0.82). As a result of analyzing the random split-half reliability, the Cronbach’s α for the entire scale was 0.90, while each factor ranged 0.81–0.84 (Table 4). 

### 3.8. Final Scale

The final scale consisted of 19 items over three dimensions including professional situations (10 items), advocating situations (4), and caring situations (5). Each item was rated on a five-point Likert scale; a higher score indicates higher nursing profession self-efficacy.

## 4. Discussion

This study tested the validity and reliability of the K-NPSE in an attempt to develop a nursing profession self-efficacy scale suitable to Korean culture. It is important to present reasonable evidence with various logical analyses for using the scale [15,24]. Thus, we recruited nurses from multiple regions in Korea in consideration of the features of the clinical setting and adhered to systematic procedures. 

In the presence of cultural differences, translated scales need to be verified for their content validity [25]. Thus, this study was followed to WHO’s guideline for translation and application of a scale [16] starting from the translation stage. Six nursing experts reviewed the content validity over two rounds of analysis, and a pilot test was performed on nurses to assess cognitive validity, whereby the contents and comprehension of the items were evaluated. The resulting K-NPSE scale, therefore, contains appropriate contents to measure nursing professionals’ self-efficacy in the Korean clinical setting.

After performing EFA, CFA, convergent validity testing, and discriminant validity testing to establish the construct validity of the scale, the K-NPSE with three factors was finalized. While the original scale had two dimensions, the K-NPSE was further divided into three factors without hurting the theoretical construct of the original scale. Because social norms and acceptance may differ across cultures and generations, items can be moved and factors divided [26]. Thorough review of the construct in NPSE, the three factors of the K-NPSE were named as “professional situation,” “advocating situation,” and “caring situation,” respectively.

In the “professional situation,” all of the items that had been included in the “professional situation” of the NPSE were included. Despite the different culture, it seemed to similar stances toward professional situations in nursing settings. Items number 6 (ethical dilemmas related to caring work), 8 (quality of clinical documentation), and 12 (ethical/moral dilemmas and problems) for the first dimension of the NPSE, “characteristics of nursing situation,” were included in the “professional situation” in the K-NPSE. This result seemed to reflect the recent emphasis on ethics and social responsibilities among nursing professionals [27,28]. 

From 12 items that had been included in the “characteristics of nursing situation” in the original scale, the remaining 9 items were classified into the “advocating situation” and “caring situation” factors in the K-NPSE. Advocacy is one of important roles for professional nurses [27,28], because advocacy among nurses refers to their effort to preserve the fundamental values of patients, such as dignity and privacy, and to protect patients from harmful behaviors [2]. Therefore, items number 7 (respect for professional confidentiality), 9 (legal and moral rights of patients), 10 (patients’ privacy), and 11 (fair use of the resources) which contain contents related to nurse advocating activities, were included in the “advocating situation.” Therefore, the result that advocacy as a subcategory of K-NPSE was derived is considered very meaningful in this study.

The third factor of the K-NPSE, the “caring situation,” consisted of five items (1; patient’s autonomy, 2; safety of society, 3; professional standards, 4; individualized health care, and 5; compensating for the shortcomings and inefficiencies). Nurses take care of patients directly or help patients and their family to resolve the issues on their own [6]. This factor of K-NPSE related to nursing performance was considered to reflect the unique tasks of nursing professionals.

The item analysis was examined using the mean, standard deviation, skewness, and kurtosis of the K-NPSE items and these score distributions were stable. Furthermore, the quality of the items was also established, as ITC was above 0.30 [14] and Cronbach’s α with items deleted was consistent. Criterion-related validity was also established. However, the correlation between the advocating situation of K-NPSE and general self-efficacy was a little low. It seemed to suggest that the nurse’s role as an advocate cannot be explained by general self-efficacy and that nurses have a special role as professionals. Therefore, the K-NPSE is a scale that can more accurately measure nursing professionals’ self-efficacy. 

In this study, internal consistency was examined using Cronbach’s α to establish the reliability of the K-NPSE, and the reliability was established even with a small sample. The Cronbach’s α for the K-NPSE was 0.93, and that for each factor was also high, above 0.82. Further, split-half reliability was high at 0.90, and that for each factor was also moderate at above 0.81. These results confirm that the K-NPSE is a reliable scale for measuring nursing profession self-efficacy. 

There are growing demands for quality nursing services, and nurses are required to be equipped with the professional knowledge and skills necessary for dealing with various situations that can occur in complex nursing settings [11,12]. Considering the professional work and unique roles of nurses, accurately measuring their professional self-efficacy is crucial. This study validated a K-NPSE scale which is better suitable for nursing professional self-efficacy due to the detailed construct of three factors, and it is expected to contribute to clinical nursing practice and research in the future. In comparison with the original NPSE, which reflects the Italian nursing culture, none of the items were dropped or deleted in the K-NPSE, but the composition of structure was changed. The K-NPSE scale will be useful to assess clinical nurses’ professional self-efficacy.

## 5. Conclusions

This study validated the Korean version of a scale measuring the NPSE. The validity and reliability of the 3-dimension, 19-item K-NPSE was established. The K-NPSE may be used as a valuable reference for developing programs or policies that promote nursing professionals. Furthermore, this scale will be useful in research that measures clinical nurses’ professional self-efficacy. It is expected that continued use of this scale in various clinical settings will further generalize and validate the scale.

## Figures and Tables

**Table 1:** Exploratory Factor Analysis of K-NPSE (N = 298).

No.	Item	Commu-nalities	Factor Structure		
			1	2	3
15	I have confidence to implement the results of research in professional practice	0.62	0.76	0.08	0.17
17	I have confidence to take part in nursing research	0.60	0.76	0.04	0.16
18	I have confidence to collaborate with nursing organizations to ensure the best standards of care in my practice	0.63	0.73	0.28	0.16
16	I have confidence to refuse to participate in treatment if is contrary to professional values	0.52	0.67	0.19	0.20
13	I have confidence to base my work on scientifically validated and updated knowledge	0.48	0.63	0.17	0.25
8	I have confidence to examine the quality (accuracy/completeness) of clinical documentation	0.48	0.58	0.15	0.35
12	I have confidence to practicing the profession, recognizing and addressing the ethical/moral dilemmas and problems of everyday working life	0.57	0.58	0.44	0.21
6	I have confidence to promote the use of ethics consultation for ethical dilemmas related to caring work	0.55	0.57	0.06	0.46
19	I have confidence to report any abuse or unethical behavior of colleagues to the appropriate Regulatory Authority/Body	0.42	0.47	0.43	0.09
14	I have confidence to use the support of other colleagues to evaluate a particular situation or problem	0.43	0.46	0.41	0.21
10	I have confidence to safeguard the right of patients’ privacy and confidentiality in data processing	0.81	0.05	0.88	0.18
7	I have confidence to promote respect for professional confidentiality	0.71	0.08	0.80	0.23
9	I have confidence to safeguard the legal and moral rights of patients	0.67	0.32	0.69	0.30
11	I have confidence to ensure the fair use of the resources that I have in my professional practice	0.60	0.41	0.62	0.22
2	I have confidence to Safeguard health and the safety of society	0.66	0.33	0.17	0.72
4	I have confidence to deliver individualized health care, based on the principle of equity and provided without discrimination or prejudice	0.58	0.21	0.25	0.69
3	I have confidence to ensure health care is delivered in line with professional standards, regardless of any singular situation	0.64	0.34	0.24	0.68
1	I have confidence to respect patients and their autonomy(e.g., principles of freedom of choice or self-determination)	0.61	0.01	0.43	0.65
5	I have confidence to compensate for the shortcomings and inefficiencies that may occur in the facility where I work	0.57	0.48	0.15	0.57
	Explained variance		4.74	3.35	3.05
	Explained %		24.9	17.7	16.1
	Cumulative %		24.9	42.6	58.7

K-NPSE = Korean version of the Nursing Profession Self-Efficacy scale.

**Table 2:** Confirmatory Factor Analysis of K-NPSE (N = 218).

No.	Estimate	SE	FL	CR	*p*	CR	AVE
Factor 1: Professional situation							
15	1.00	-	0.70	-	-	0.91	0.51
17	0.87	0.11	0.59	8.06	<0.001		
18	0.97	0.10	0.70	9.42	<0.001		
16	0.82	0.10	0.62	8.44	<0.001		
13	0.68	0.09	0.58	7.85	<0.001		
8	0.82	0.10	0.59	8.04	<0.001		
12	0.78	0.09	0.64	8.63	<0.001		
6	0.82	0.10	0.63	8.52	<0.001		
19	0.80	0.11	0.53	7.26	<0.001		
14	0.67	0.09	0.55	7.50	<0.001		
Factor 2: Advocating situation							
10	1.00	-	0.84	-	-	0.93	0.77
7	1.03	0.08	0.82	13.68	<0.001		
9	0.94	0.08	0.76	12.41	<0.001		
11	0.86	0.08	0.72	11.47	<0.001		
Factor 3: Caring situation							
2	1.00	-	0.72	-	-	0.89	0.62
4	0.97	0.11	0.67	9.01	<0.001		
3	1.09	0.11	0.76	10.18	<0.001		
1	0.78	0.09	0.63	8.59	<0.001		
5	1.06	0.12	0.68	9.14	<0.001		
Model Fitness		χ^2^ = 398.91, *p* < *0*.001, χ^2^/df = 2.68, GFI = 0.85, AGFI = 0.80, CFI = 0.86, NFI = 0.80, TLI = 0.84, RMSEA = 0.09					

AGFI = Adjusted Goodness of Fit Index; AVE = Average Variance Extracted; CFI = Comparative Fit Index; CR = Construct reliability; FL = standardized Factor Loading; GFI = Goodness of Fit Index; K-NPSE = Korean version of the Nursing Profession Self-Efficacy; NFI = Normed Fit Index; RMSEA = Root Mean Squared Error of Approximation; SE = Standardized Estimates; TLI = Tuker–Lewis Index.

**Table 3:** Correlation Matrix among Factors of K-NPSE and General Self-efficacy (N = 218).

Variable	Factor 1	Factor 2	Factor 3	K-NPSE
	r (*p*)	r (*p*)	r (*p*)	r (*p*)
Factor 1: Professional situation	0.71 *^1^*			
Factor 2: Advocating situation	0.62 (<.001)	0.88 *^1^*		
Factor 3: Caring situation	0.66 (<0.001)	0.63 (<0.001)	0.79 ^1^	
General Self-efficacy (*N* = 298)	0.42 (<0.001)	0.26 (<0.001)	0.41 (<0.001)	0.43 (<0.001)

^1.^ square root AVE; AVE = Average Variance Extracted; K-NPSE = Korean version of the Nursing Profession Self-Efficacy.

**Table 4:** Item Analysis and Reliability of K-NPSE (N = 298).

Item No.	Mean	SD	SE	Skewness	Kurtosis	ITC	α If Item Deleted	Mean	SD	Cronbach’s α	
										Total (*n* = 298)	Half (*n* = 149)
K-NPSE								71.23	8.65	0.93	0.90
Factor 1: Professional situation								36.53	5.06	0.88	0.84
15	3.58	0.71	0.04	−0.19	−0.16	0.61	0.92				
17	3.52	0.78	0.05	−0.06	0.04	0.58	0.92				
18	3.66	0.76	0.04	−0.31	0.35	0.68	0.92				
16	3.64	0.76	0.04	−0.40	0.40	0.61	0.92				
13	3.68	0.63	0.04	−0.05	−0.18	0.60	0.92				
8	3.64	0.73	0.04	−0.04	−0.29	0.61	0.92				
12	3.81	0.68	0.04	−0.24	0.06	0.68	0.92				
6	3.43	0.69	0.04	0.15	−0.17	0.61	0.92				
19	3.72	0.81	0.05	−0.37	−0.05	0.54	0.92				
14	3.85	0.65	0.04	−0.21	0.12	0.58	0.92				
Factor 2: Advocating situation								16.17	2.17	0.85	0.81
10	4.13	0.64	0.04	−0.21	−0.30	0.52	0.92				
7	4.14	0.68	0.04	−0.31	−0.35	0.53	0.92				
9	3.94	0.67	0.04	−0.07	−0.39	0.68	0.92				
11	3.96	0.62	0.04	−0.14	0.12	0.66	0.92				
Factor 3: Caring situation								18.54	2.57	0.82	0.81
2	3.66	0.66	0.04	0.17	−0.39	0.64	0.92				
4	3.83	0.71	.04	−0.35	0.14	0.58	0.92				
3	3.82	0.64	0.04	−0.20	0.14	0.67	0.92				
1	3.80	0.60	0.03	−0.00	−0.20	0.51	0.92				
5	3.44	0.74	0.04	−0.08	−0.33	0.65	0.92				

ITC = Item-Total Correlation; K-NPSE = Korean version of the Nursing Profession Self-Efficacy; SD = Standard Deviation; SE = Standard Error.

## Data Availability

The data presented in this study are available on request from the corresponding author. The data are not publicly available due to privacy.
